# Evaluation and comparison of antibacterial efficacy of different concentrations of Chhattisgarh herbal product—*Terminalia chebula* fruit extract in opposition to *Enterococcus faecalis*: An in vitro study

**DOI:** 10.1002/fsn3.3814

**Published:** 2023-11-29

**Authors:** Shivani Deepak Doye, Manjunath Malur, Yogesh Sahu, Ankita Singh, Praveen Mishra, Roshan Noor Mohamed, Mohmed Isaqali Karobari

**Affiliations:** ^1^ Department of Conservative Dentistry and Endodontics Maitri College of Dentistry and Research Centre Anjora, Durg Chhattisgarh India; ^2^ Department of Pediatric Dentistry, Faculty of Dentistry Taif University Taif Saudi Arabia; ^3^ Department of Restorative Dentistry and Endodontics, Faculty of Dentistry University of Puthisastra Phnom Penh Cambodia; ^4^ Dental Research Unit, Center for Global Health Research Saveetha Institute of Medical and Technical Sciences University Chennai Tamil Nadu India

**Keywords:** antibacterial activity, dental, endodontics, *Enterococcus faecalis*, herbal product, *Terminalia chebula* extract

## Abstract

Due to the low cost, natural origin, higher safety margins, and little to negligible adverse effects of herbal medications, the use of plants and plant derivatives in medicine is becoming increasingly widespread. *Terminalia chebula* is among the most significant medicinal plants in ayurvedic, siddha, unani, and homeopathic remedies. It is ranked first in Ayurvedic material medicine. *T. chebula* has been shown to have established effects against various bacterial and fungal infections, including dental caries pathogens. In recent years, there has been a rise in interest in dentistry and medicine related to *Enterococcus faecalis*. The research aimed to assess the antibacterial effectiveness of different concentrations of *T. chebula* ethanolic fruit extract (10%, 40%, and 100%) in opposition to *E. faecalis* and compare it with 2% chlorhexidine. For the study, *T. chebula* ethanolic fruit extracts were obtained and prepared with Group I: −10% concentration, Group II: −40% concentration, Group III: −100% concentration, and Group IV: −2% chlorhexidine. Colonies of *E. faecalis* were cultivated in brain heart infusion (BHI) broth at 37°C and were inoculated in 16 BHI agar plates. Then, on the petri dishes, four wells were created (8 mm diameter) using a metal borer. The Agar well diffusion method was used to examine the antibacterial activity, and the zones of inhibition around the wells were noted. The obtained data were statistically analyzed using one‐way ANOVA and post‐hoc tests. The result shows that as the concentration increases, there is an increase in the efficacy of the antibacterial property of the extract before it reaches the saturation point. The decreasing order of antibacterial was chlorhexidine >100% *T. chebula* >40% *T. chebula* >10% *T. chebula*. The production of contemporary pharmaceuticals from *T. chebula* was addressed, as the global scenario is currently evolving toward using nontoxic plant products with traditional medicinal benefits.

## Graphical abstract

The ethanolic extract from *Terminalia chebula* fruit was effective, as discovered against *E. faecalis* within the confines of the present study. Further, we can say that the antibacterial property of the extract is directly proportional to its concentration up to the saturation point.

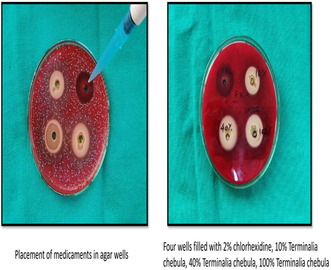

## 1 INTRODUCTION

Due to the negligent utilization of currently available antimicrobial medications in managing infectious disorders, diverse drug resistance has recently emerged (Ahmed et al., 2022). It is essential to renew our work in this area to defeat infectious agents that are resistant to conventional antibiotics. Furthermore, even though conventional antibiotics are powerful medicines that help save lives when not used correctly, they have more significant adverse effects than beneficial ones (Ramzan et al., 2007). As a consequence, it is also necessary, in comparison to other sources, to improve alternative antimicrobial agents in agents to treat microbial infections. According to the World Health Organization, 80% of the worldwide population primarily utilizes traditional medicines that involve plant extracts or their active constituents (Afzal et al., 2023; Tariq et al., 2020).

To preserve plant resources in their natural state, the Chhattisgarh government designated the state as a “Herbal State.” Due to suitable agro‐climatic circumstances, such as adequate rainfall and very little biotic intervention, Chhattisgarh has roughly 44% of its geographical area covered by forests, which is exceptionally rich in biodiversity (Nayak et al., 2014). The state features extensive forested areas with all three canopies. Rural populations use these items as medicines and dietary supplements and profit significantly from collecting and selling these goods, particularly outside of the agricultural season (Prabhakar et al., 2010; Rai & Joshi, 2009).

One of the many herbs which can be discovered in Chhattisgarh herbals, “*Terminalia chebula*,” has been used for both the prevention and treatment of numerous illnesses since the beginning of time. The scientific names for *T. chebula* include Haritaki, Alalekayi, and Harad due to its exceptional therapeutic abilities (Nayak et al., 2014). *T. chebula* is called the “King of Medicine” in Tibet and is consistently named first in the Ayurvedic Materia Medica. Asthma, sore throat, vomiting, hiccoughing, diarrhea, bleeding piles, gout, and heart and bladder disorders have all been traditionally treated with the dried, ripe fruits of Chebulic myrobalan. It exhibits anticarcinogenic, antioxidant, and free radical scavenging activities (Bag et al., 2009). According to research on *T. chebula*, it has a broad spectrum of antibacterial, antiviral, antifungal, cytoprotective, hepatoprotective, and radioprotective effects (Nayak et al., 2014).

In the past decade, there has been a rise in interest in dentistry and medicine related to *Enterococcus faecalis* (Assiry et al., 2023; Karobari et al., 2022) and several herbal fruit extracts (Al‐Musawi et al., 2022; Khudier et al., 2023). It is challenging to eradicate a pathogen from a root canal utilizing chemomechanical preparation, disinfecting irrigants, and antibacterial dressings in post‐treatment endodontic infection (Ahmed et al., 2022; Kandaswamy & Venkateshbabu, 2010).

Extracts from *T. chebula* have been shown to have antibacterial effects on several bacterial strains. It effectively inhibits *Helicobacter pylori*, *Xanthomonas*, and *Salmonella typhi* (Chattopadhyay & Bhattacharyya, 2007a; Gupta, 2012). In light of these alleged medical benefits, the current experiment evaluated the antibacterial ability of different concentrations of *T. chebula* ethanolic fruit extract in opposition to *E. faecalis* and compared its effectiveness with 2% chlorhexidine. Numerous studies have been done on the antibacterial activity of *T. chebula* against various pathogens, but there are very few works of literature on concentrations of *T. chebula* used against *E. faecalis*; hence, this study has been done for its antibacterial activity against most persistent bacteria *E. faecalis* adding the concentration as a variable. This will help explore the potential use of this herbal medicine in the field of dentistry.

## 2 MATERIALS AND METHODS

The *T. chebula* extract was commercially available at the Chhattisgarh herbal sanjeevani shop and was collected and used in the current study. The ethical approval was waived off by the human ethics committee of Maitri College of Dentistry and Research Centre, Anjora, Durg, Chhattisgarh, due to the involvement of humans in the current research. The sample size calculations were carried out using the maximum standard deviation and the precision of marginal interval calculated by a previously conducted study by Grover et al. (2016). A total of 16 agar plates were inoculated to assess the antibacterial effectiveness of *T. chebula*.

### 2.1 Preparation of extract

The *T. chebula* dried fruit powder was collected from the Chhattisgarh herbal sanjeevani shop, Durg. The powder was then used to prepare the ethanolic extract in three different concentrations:

1. 10% concentration − 10 g of powder in 100 mL of ethanol
2. 40% concentration – 40 g of powder in 100 mL of ethanol
3. 100% concentration – 100 g of powder in 100 mL of ethanol

The powder was soaked in 99% ethanol for three days at room temperature to prepare the extract in respective concentrations. Then, the solutions were concentrated using the fractional distillation method.

### 2.2 Culture of *E. faecalis*

*E. faecalis* strains were inoculated in brain heart infusion (BHI) broth and incubated at 37°C for 24 h. Then, the growth of the bacteria was confirmed by the changes in the turbidity of the broth. BHI broth, inoculated with *E. faecalis*, was then used to inoculate 16 agar plates. Cultures were transferred from broth to 16 agar plates and incubated for 24 h by pour plate method. The uniform lawn of *E. faecalis* was seen on the agar plates.

### 2.3 The procedure of placing the medications

**FIGURE 1:**
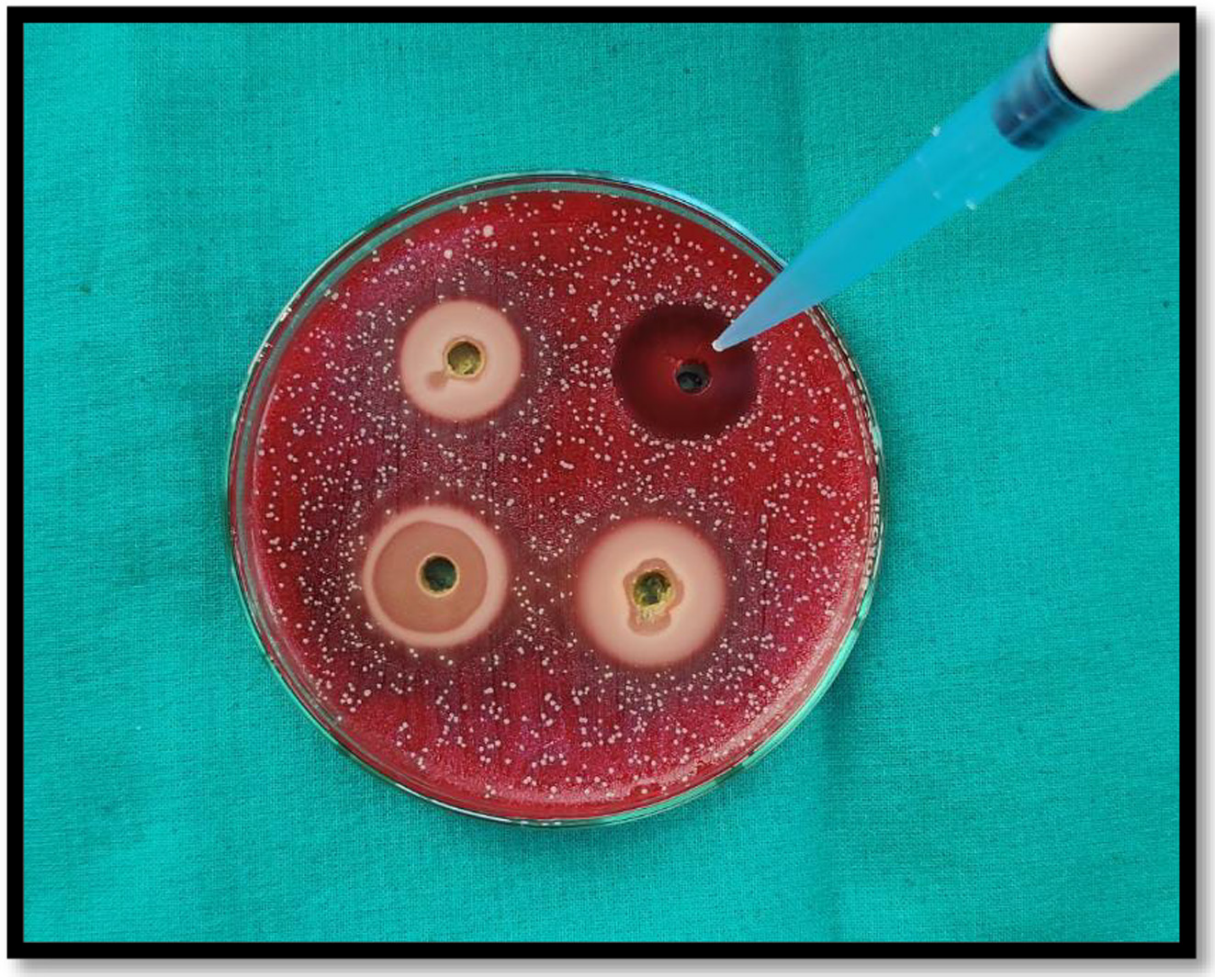
Placement of medicament in agar wells.

**FIGURE 2:**
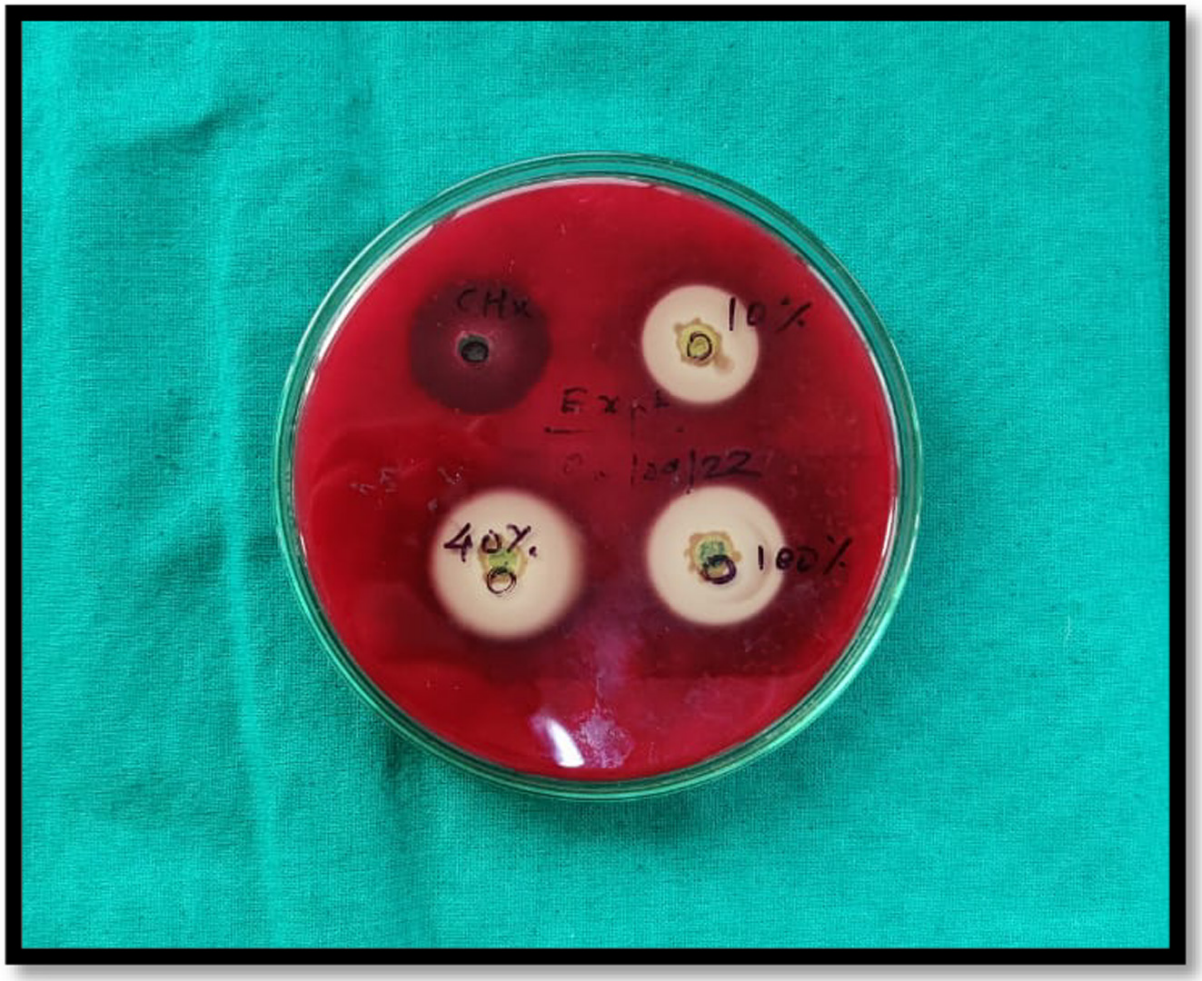
Four wells filled with 2% chlorhexidine, 10% *Terminalia chebula*, 40% *T. chebula*, 100% *T. chebula*.

In the agar well diffusion method, wells are created on the agar, and medicament is diffused to check its activity. Using a metal borer, four 8‐mm‐diameter wells were created on Petri dishes. Using a micropipette, the material was added to these created wells. Out of four wells, one was filled with 2% chlorhexidine, 10% *T. chebula*, 40% *T. chebula*, and 100% *T. chebula*, respectively (Figures 1 and 2). Then, incubation of medicated petri dishes was done for 24 h at 37°C.

### 2.4 Zone of inhibition

The zones of Inhibition emerged around each wall as per the antibacterial activity of the medications. Then, a ruler measured the clear area's diameter (in mm), while the lid was still in place. To prevent contamination, all procedures were carried out in laminar airflow.

### 2.5 Statistical analysis

One‐way analysis of variance was applied to find the difference between the zone of Inhibition within and between groups (*p* < .001). Post‐hoc tests were applied to make multiple comparisons between different groups.

## 3 RESULTS AND DISCUSSION

Recent years have seen a considerable increase in the importance of green technology in the research community since it is easy to use, nontoxic, less time‐consuming, less expensive, and can be produced on a colossal scale (Al‐Radadi et al., 2022). The antibacterial activity was evaluated by measuring the zones of inhibition. The values obtained were analyzed using (Table 1) one‐way ANOVA and post‐hoc tests. The decreasing order of antibacterial efficacy observed was chlorhexidine > 100% *T. chebula* > 40% *T. chebula* > 10% *T. chebula* (Table 2).

**TABLE 1:** Comparison of zone of inhibition (mean) in mm among Group A (chlorhexidine), Group B (10% *Terminalia chebula*), Group C (40% *T. chebula*), and Group D (100% *T. chebula*) after applying ANOVA test.

S.N.	Groups	*N*	Zone of inhibition (mm) (mean ± SD)	*F* value	*p*‐value
1.	Group A (chlorhexidine)	16	16.0 ± 1.09	33.58	0.000 (*p* < .001) Very highly significant
2.	Group B (10% *T. chebula*)	16	10.06 ± 2.54		
3.	Group C (40% *T. chebula*)	16	14.31 ± 1.50		
4.	Group D (100% *T. chebula*)	16	14.62 ± 1.62		

**TABLE 2:** Multiple pairwise comparison of mean zone of inhibition in mm after applying ANOVA test among Group A (chlorhexidine), Group B (10% *Terminalia chebula*), Group C (40% *T. chebula*), and Group D (100% *T. chebula*) where (S)—significant, (NS)—not significant, (HS)—highly significant.

Groups comparison	Mean difference	*p*‐value
Group A (chlorhexidine)		
Group B (10% *T. chebula*)	5.93750*	.000 (<.001) (HS)
Group C (40% *T. chebula*)	1.37500	.044 (<.05) (S)
Group D (100% *T. chebula*)	1.68750*	.136 (>.05) (NS)
Group B (10% *T. chebula*)		
Group A (chlorhexidine)	−5.93750*	.000 (<.001) (HS)
Group C (40% *T. chebula*)	−4.56250*	.000 (<.001) (HS)
Group D (100% *T. chebula*)	−4.25000*	.000 (<.001) (HS)
Group C (40% *T. chebula*)		
Group A (chlorhexidine)	−1.68750*	.044 (<.05) (S)
Group B (10% *T. chebula*)	4.25000*	.000 (<.001) (HS)
Group D (100% *T. chebula*)	−.31250	.959 (>.05) (NS)
Group D (100% *T. chebula*)		
Group A (chlorhexidine)	−1.37500	.136 (>.05) (NS)
Group B (10% *T. chebula*)	4.56250*	.000 (<.001) (HS)
Group C (40% *T. chebula*)	.31250	.959 (>.05) (NS)

* significant diffrence.

The *T. chebula* is a member of the Combretaceae family. It has earned the title of “King of Medicines.” It is recognized to have tremendous medicinal power because it is thought to have come from Ambrosa. Its fruit, bark, and leaves are frequently used for medicinal purposes (Chattopadhyay & Bhattacharyya, 2007b; Sheng et al., 2023). Tannins, anthraquinones, and polyphenolic substances are present in the fruit. Tannins were previously used to treat oral inflammatory disorders (Nayak et al., 2014).

Numerous investigations have focused on finding a practical means of eliminating *E. faecalis* from the root canal space and/or preventing it from doing so. *E. faecalis* can enter the root canal system while undergoing treatment, in between visits, or even following the end of the procedure. Therefore, it is crucial to consider treatment plans that attempt to eliminate or prevent the *E. faecalis* infection during each period (Stuart et al., 2006).

Due to its well‐established efficacy in eradicating *E. faecalis*, 2% chlorhexidine was chosen as a test specimen in the current study. Because of its well‐known effects, chlorhexidine has been employed as an experimental sample in several comparative evaluations to assess the effectiveness of more recently investigated materials (Grover et al., 2016). The comparison has been made by measuring the zone of Inhibition of all the samples. The zone of Inhibition of Group A (chlorhexidine) was 16.0 ± 1.09, while Group D (100% *T. chebula*) was found to be 14.62 ± 1.62. There was no significant difference between the antibacterial activity of chlorhexidine and 100% *T. chebula*. This suggests that *T. chebula* can be used as an herbal alternative to chlorhexidine. This herbal alternative will show no side effects and will be safer.

The biological influence of plant extracts is caused by their naturally occurring, chemically complex components, which can be employed singly or in combination (Al‐Musawi et al., 2022). Fruit from the *T. chebula* plant was used for the study because of its high tannin content (approximately 30–40% tannin). It has been claimed that tannins have strong antibacterial properties (Afzal et al., 2023). The ability of tannins to penetrate the bacterial cell wall and reach the interior membrane, their interference with the cell's metabolism, and—as a result—their destruction account for their antibacterial activity (Al‐Musawi et al., 2022). It has also been employed because several phytochemical components have been associated with the plant extract, primarily the various forms of chebulic acid, gallic acid, ellagic acid, tannins, amino acids, and flavonoids. Many pharmacological functions have been attributed to these compounds (Nayak et al., 2014; Raval et al., 2023).

Different extracts of *T. chebula* show varying degrees of antibacterial potential against *E. faecalis*, out of which ethanolic extract showed superior activity, and most of the tannins are soluble in ethanol. Ethanol was used as a solvent in the present study (Bag et al., 2009). *Staphylococcus*, *Staphylococcus epidermidis*, and *Bacillus subtilis* are just a few of the bacterial species that the ethanol extract of *T. chebula* fruit has been shown to be effective against in numerous studies. These results point to the beneficial effects of the antibacterial or antiseptic activities of *T. chebula* fruit (Kannan et al., 2009; Sumathi & Parvathi, 2010).

The antibacterial study suggests that different concentrations (10%, 40%, and 100%) of fruit extract had efficient antibacterial activity in opposition to *E. faecalis*. 40% concentration of the extract showed more significant antibacterial effectiveness than the extract of 10%. On the other hand, the difference between the zone of inhibitions of 40% and 100% concentration showed no significant difference.

To summarize all this, we can say that as the concentration increases, there is an increase in the efficacy of the antibacterial property of the extract before it reaches the saturation point. The present study's concentration of prepared herbal solutions was directly proportional to their antibacterial activity. The reason could be due to less solubility of the active constituents after a substance can no longer be dissolved, absorbed, or otherwise combined with solvent. Herbal solutions typically target the planktonic cells than the sessile bacteria. This could be why the different concentrations of herbal alternatives affect the antibacterial activity of the solution.

Comparing the zone of inhibition of 100% concentration extract to standard disinfectant (chlorhexidine) showed comparatively similar activity. Hence, it can be suggested as an alternative to synthetic agents. As the present research was carried out in vitro, there might be changes in the reading inside the oral environment. *T. chebula* shows antibacterial activity against many pathogens, and there might be changes in the values if other bacterial strains were used. Also, for more specific quantitative data, further research has to be done for the critical numerical and specific formulas to reinforce the observed activity.

Nanotechnology is now recognized as an established cutting‐edge technology with many applications in the pharmaceutical and other industries (Faisal et al., 2021). *T. chebula* also has a chance to be studied in the field of nanomedicines.

## 4 CONCLUSION

The ethanolic extract from *T. chebula* fruit was effective, as discovered against *E. faecalis* within the confines of the present study. Further, we can say that the antibacterial property of the extract is directly proportional to its concentration up to the saturation point. It has been studied that generations of parachloroanilline and reactive oxygen species in chlorhexidine are possibly carcinogenic. The constant increase in antibiotic‐resistant strains and side effects caused by synthetic drugs has prompted research outlook in the direction of herbal alternatives. As nontoxic plant items with historical uses for medicine are becoming more prevalent worldwide, additional research is advised to further characterize active principles and manner of application to maximize the observed activity and, consequently, their potential utility in endodontics.

## AUTHOR CONTRIBUTIONS

**Shivani Deepak Doye:** Conceptualization (equal); data curation (lead); investigation (lead); writing – original draft (lead). **Manjunath Malur:** Conceptualization (equal); data curation (supporting); investigation (supporting); methodology (equal); project administration (equal); writing – original draft (supporting). **Yogesh Sahu:** Validation (lead); writing – original draft (supporting). **Ankita Singh:** Validation (supporting); writing – review and editing (supporting). **Praveen Mishra:** Formal analysis (supporting); writing – review and editing (supporting). **Mohmed Isaqali Karobari:** Conceptualization (lead); methodology (equal); project administration (equal); supervision (lead); writing – original draft (supporting); writing – review and editing (lead).

## FUNDING INFORMATION

This research received no external funding.

## CONFLICT OF INTEREST STATEMENT

The authors declare no conflict of interest.

## INSTITUTIONAL REVIEW BOARD STATEMENT

This study does not involve any human or animal testing.

## CONSENT FOR PUBLICATION

All listed authors have read the final manuscript and provided consent for publication.

## Data Availability

The data will be made available on reasonable request to the corresponding author.
